# Stunting and thinness in school-attending adolescents in Addis Ababa

**DOI:** 10.1186/s40795-022-00653-1

**Published:** 2022-12-30

**Authors:** Walelegn Worku Yallew, Amare Worku Tadesse, Abdallah Noor, Wafaie Fawzi, Yemane Berhane

**Affiliations:** 1 Addis Continental Institute of Public Health, Po. Box 196, Addis Ababa, Ethiopia; 2 Department of Infectious Diseases Epidemiology, London School of Hygiene and Tropical Medicine, London, UK; 3 United Nations Children’s Fund, New York, USA; 4 Department of Nutrition, Harvard TH Chan School of Public Health, Boston, MA USA

**Keywords:** School adolescents, Thinness, Stunting, Global diet quality score, Ethiopia

## Abstract

**Background:**

Adolescent population Global Diet Quality Score (GDQS) is uncommon in low-income settings. Though Global Diet Quality Score is a good measure of dietary diversity, it has not been used in assessing nutritional outcomes among adolescents. Therefore, the aim of this study is to assess school-attending adolescents stunting and thinness status and associations with global diet quality scores in Addis Ababa.

**Methods:**

A school-based cross-sectional study was conducted among school-attending adolescents in the age group of 10–14 years in urban settings in Ethiopia. A multi-stage stratified random sampling procedure was used to select schools and students. A total of 1200 adolescents were included in the study. Binary and Multinomial logistic regression analyses were used to determine the predictors of stunting and thinness respectively.

**Results:**

The proportion of school-attending adolescents stunting was 8.17% (95% CI: 6.74%,9.85%) and their thinness status 12.66% (95% CI: 10.89%, 14.67%). The overall mean GDQS food groups for Seven days period consumed were 19.99 + 2.81 SD. Male adolescents were 1.95 times more likely to be stunted compared to female adolescents (1.95; 95%CI: 1.11,3.39). Frequent consumption of low-fat dairy increased the risk of thinness, while frequent consumption of citrus fruits and white root tubers decreased the risk of school-attending adolescents’ thinness.

**Conclusion:**

The proportion of thin or stunted adolescents attending school was high still, about 1 in 10. Stunting and thinness have no association with the overall GDQS. Nutritional interventions need to consider frequent consumption of citrus fruits, low-fat dairy, and white roots and tubers in school adolescents’ nutritional programs. Further studies should validate the GDQS for stunting and thinness of school adolescents.

## Background

Adolescents are vulnerable to malnutrition because of rapid growth and development and their high macro and micronutrient demand [1]. Adolescence is also a window of opportunity for establishing lifelong dietary habits that support nutritional well-being of generations [2]. The burden of malnutrition in developing countries among adolescents is high [3]. In Ethiopia, a significant number of school adolescents are affected by malnutrition [4]. Researches in Addis Ababa showed that 15. 15.9% of school age children are underweight [5]. In Ethiopia, stunting prevalence among school adolescents range from 5–29% [4, 6–8]. Risk factors for stunting include mothers’ occupation [7, 8], mothers’ education [7], adolescents age [6, 7] and grade [9], dietary diversity score [6], meal frequency [9], and food insecurity status [9]. In addition, in Ethiopia, most adolescents are dependent on their parents. Therefore, parents’ education and family income have an influence on adolescent nutritional status [10, 11]. Culturally boys had more free time to participate in outdoor physical activities than adolescent girls; which influence their body weight [12].

Studies indicate that thinness prevalence among school adolescent ranges from 4.5–29% [6–8, 13, 14]. In low-income countries, adolescents thinness is influenced by their parents education level [7], age of the adolescent [8, 14], family income [14], dietary diversity score [6], frequency of meal [6] and residency [8]. In addition, exposure of school adolescents to unhealthy food consumption [15] around school environment may have effect on their nutritional status. Adolescents’ food choices is based on peer pressure other than nutritional composition and adolescents may perceive either excess weight or thinness as a sign of well-being and attractiveness [16, 17]. The effect is eating disorders during adolescence [18].

Research recommend a need for consistently measured and standardized indicators for dietary diversity [19]. Dietary diversity is one dimension of diet quality. There are different type of dietary quality assessment methods [20, 21]. Global Diet Quality Score (GDQS) is one of the recent methods to capture both under- and overnutrition [22]. The GDQS was designed for non-pregnant, non-lactating women of reproductive age in low- and middle-income countries (LMICs) [23–25]. The method was tested for mothers but has not been tested for adolescents [20, 22, 26, 27]. Focusing on adolescents’ populations will benefit present and future generations in terms of health and development.

Dietary diversity practice has an association with underweight for school adolescents [28]. Poor dietary diversity and food variety are directly associated with inadequate micronutrient intake [29]. A review in low- and middle-income countries show that daily consumption of nutritious foods is low for school adolescents. On average, 46% adolescents consume daily meat, 44% fruits, and 37% vegetables [19].

Although there are improvements in nutritional problem in Ethiopia [30], the government is still off-track to achieve nutritional targets at 2025 [31]. This may help to develop nutrition plans for health and development program for this age group among national priorities. Therefore, school adolescent nutritional status assessment is very important. The aim of this study is to assess stunting and thinness in school-attending adolescents in Addis Ababa.

## Methods

### Design and setting

A cross-sectional study was conducted among school-attending adolescents aged 10–14 years. The study was conducted in Addis Ababa, the capital city of Ethiopia. Administratively the city is divided in to 10 sub-cities. Addis Ababa is the largest and one of the fastest growing cities in the continent. A total of 223 primary government school exists in the city. The survey was conducted from May to June 2019 in 20 primary schools. Schools in subcities such as Addis Ketema, Akaky Kaliti, Arada, Bole, Gullele, Kirkos, Kolfe Keraino, Lideta, Nifas Silk-Lafto, and Yeka were included.

### Participants

Sample size was calculated assuming stunting prevalence of 7.2% [32], level of significance 2%, design effect of 1.5, 20 school clusters and 10% non-response rate. A total of 1078 sample size was calculated to get adequate power to answer our objective. While we have taken all 1200 students collected information to the project to acquire adequate precise information from the available data. A multi-stage stratified random sampling procedure was used to select schools and students. First, randomly two government schools were selected from each sub-city. A total of 20 schools were included in the study. From each selected school, randomly 15 students enrolled whose age were 10–14 years old from each of the grades 5–8. A total of 60 students per school were enrolled to the study. An updated student roster was used as a sampling frame to select students. A total of 1,200 school-attending adolescents were included in the study.

### Variables measurement

#### Outcome’s definitions

Stunting and thinness were assessed according to WHO definition for adolescent. Height was measured in meters to the nearest centimetre. Weight was measured using portable standing digital scale. Measurements were transformed into height-for-age z-scores based on sex and age in year using WHO Anthro-Plus software [33]. Height-for-age z-scores were categorized according to WHO stunting cut-offs (Stunted: <− 2SD; Not stunted: ≥ −2 SD) [34]. Thinness was defined as adolescents with BMI-for-age with Z-score < − 2 SD from the median value of WHO’s 2006 reference data [34].

#### Predictors definitions

In the study the primary exposures of interest were school adolescents Global Diet Quality Score (GDQS). Adolescents were asked food groups consumed in the reference for the past one-week period. Points were assigned for consumption of food groups based three classifications: never or 1/wk, 2–4/wk and 5–7/wk or > 1/day. Points for each food group were then summed to give an overall score. Global Diet Quality Score were recorded based on the past 7 days for 25 GDQS food groups. GDQS was classified in to two GDQS sub-metrics, such as GDQS positive (GDQS+) sub-metric and the GDQS negative (GDQS–) sub-metric diet quality outcomes [22, 26].

##### Healthy food group

The GDQS + is the total score across the 16 healthy GDQS food groups, with a possible range of 0 to 32. Healthy food groups are; Dark green leafy vegetables, Cruciferous vegetables, Deep orange tubers, Deep orange vegetables, Deep orange fruits, other vegetables, Citrus fruits, other fruits, Legumes, Nuts and seeds, Poultry and game meat, Fish and shellfish, Whole grains, Liquid oils, Low-fat dairy, Eggs. The mean GDQS + expressed the adolescent population healthy diet quality [22].

##### Unhealthy food group

The GDQS − is the total score across the 7 unhealthy GDQS food groups and the 2 GDQS food groups that are unhealthy when consumed in excessive amounts, with a possible range of 0 to 17. Unhealthy food groups are; White roots and tubers, red meat, refined grains and baked goods, Processed meat, Sugar-sweetened beverages, Sweets and ice cream, High-fat dairy, Juice, and Purchased deep fried foods. The mean GDQS- expressed the adolescent population unhealthy diet quality [22].

##### Overall GDQS

The overall GDQS is a sum of the points across all 25 GDQS food groups. The GDQS has a possible range of 0 to 49. A GDQS ≥ 23 is associated with a low risk of nutrient inadequacy, scores ≥ 15 and < 23 indicate moderate risk, and scores < 15 indicate high risk [22].

Dietary Diversity Score (DDS) was calculated based on 10-item food indicators. These food items were; starchy staples, pulses, nuts and seeds, dairy, meat, poultry and fish, eggs, dark green leafy vegetables, other vitamin-A rich fruits and vegetables, other vegetables, and other fruits. For each food item a score of “1” was given for groups consumed and “0” for not consumed over the last one week consumed. The total DDS range from 0 to 10. Those who scores ≥ 5 were good Minimum Dietary Diversity for Women (MDD-W) and < 5 were recorded insufficient MDD-W [35].

### Data management and statistical analysis

Data were collected using a pretested standard questionnaire by trained data collectors. WHO Anthro-plus was used to convert height and weight measurements to Z-scores for 5–19 years [36]. Questionnaires were programmed using ODK for electronic data collection and translated into local languages (Amharic in Ethiopia). Data collectors and supervisors were trained on research ethics, how to take consents and anthropometric measurements. Regular supervision was conducted during data collection in the field. Height and Weight were measured for each school adolescent. Height was measured to the nearest 0.1 cm and weight to the nearest 0.1 kg using a calibrated SECA weighing scale. Each subject weighed with light clothing and no footwear. All the measurements were taken twice; when necessary, any discrepancies resolved by a third measurement. The mean values were used for data analysis.

Multinomial logistic regression was conducted to investigate factors associated with adolescent nutritional status. Adolescents’ nutritional status outcome was classified as thinness, normal and overweight. Normal weight as the reference category compared to thinness and overweight. Binary logistic regression analysis was conducted to see the association between predictor variable and stunting status of school adolescents. For stunted adolescent were considered as height-for-age *Z-*score less than − 2 standard deviation of the new WHO 2007 reference population.

First, univariate analysis was conducted to identify associations between individual variables and school-attending adolescent nutritional status (Stunting and thinness). Variables in the univariate analysis with P-Value less than 0.2 were included in the final model to. control confounders. In the final model for both stunting and thinness sex, age, mother occupation, father’s education, Global diet Quality Score, Dietary Diversity score were included in the model. Clustering effect was observed using school as a cluster effect. *P*-value < 0.05 was considered as a cut-off point for statistical significance. Statistical analyses were conducted using STATA 14 software.

## Results

### Socio-demographic characteristics

A total of 1200 school-attending adolescents included in the study with a median age of 13 years with interquartile range of 12 and 14 years. The mean age of participant was 12 years ± 1.16 years standard deviations. A quarter of students 316 (26.38%) had only one sibling. For the majority of school adolescent, the education level of fathers and mothers was at the primary level (Table 1).

**Table 1:** Socio demographic characteristics of urban school-attending adolescents in Addis Ababa ,2019 Ethiopia,

Variables	Frequency	Percent (%)
**Gender**		
Male	543	45.2
Female	657	54.7
**Age in Years**		
10 years	54	4.5
11 years	188	15.6
12 years	294	24.5
13 years	344	28.6
14 years	320	26.6
**Fathers Education**		
No schooling	225	23.1
Primary	430	44.2
Secondary	251	25.8
Technical and University	67	6.9
**Mother Education**		
No schooling	62	9.4
Primary	264	39.9
Secondary	237	35.8
Technical and University	99	14.9
**Father/male guardian occupation**		
Private	487	63.3
Government workers	228	29.6
Unemployment	46	5.9
Others (Paster, religious leader)	8	1.0
**Mother/female guardian occupation**		
Private	407	37.62
Government	238	22.00
Unemployment	46	4.25
Homemaker	391	36.14
**Number of siblings (brother and Sister in the Household)**		
No sibling	145	12.1
One sibling	308	25.7
Two siblings	316	26.4
Three siblings	225	18.8
Four and Above sibling	204	17.0

#### Dietary characteristics and nutritional status

School-attending adolescents frequently (5–7 times a week) consumed wholegrains (95.25%) and liquid oils (86.58%). Poultry, fish, dark green leafy vegetable, cruciferous vegetables, deep orange fruits, deep orange tubers were rarely consumed food groups. Legumes food group were consumed 2–4 times per week by half of the students. Juice was one of the frequently consumed food groups and 94.17% of school-attending adolescents took 5–7 times per week. The second most frequently consumed unhealthy food group was sugar-sweetened beverages, 82.08% of students consumed 5–7 times per week. Except for legumes, other vegetables, low fat dairy, and liquid oils, most of the students consumed healthy food groups one time per week. Only other vegetables, whole grains, and liquid oils were consumed at least 5 days in a week. The students rarely ate red meat and high fat dairy food groups. But most frequently ate sugar sweetened beverages, sweets and ice cream, and juice (Table 2).

The overall mean score GDQS food groups consumed by school-attending adolescents one week period was 19.99 ± 2.81 Standard Deviation (SD). The one-week period mean healthy (GDQS+) sub metrics score across 16 healthy food groups was 8.81 with ± 2.86 SD. The mean score for unhealthy (GDQS-) food groups consumed within one week was 11.17 with ± 1.59 SD.

**Table 2:** GDQS food groups consumed by school-attending adolescents and their Nutritional status in Addis Ababa

**Healthy Food groups**	**Never or 1/wk** **# of students (%)**	**2–4/wk** **# of students (%)**	**5–7/wk or ≥ 1/day** **# of students (%)**
Dark green leafy vegetables	940 (78.3)	253 (21.1)	7 (0.5)
Cruciferous vegetables	1011 (84.5)	184 (15.3)	5 (0.4)
Deep orange vegetables	838 (69.3)	345 (28.7)	17 (1.4)
Deep orange fruits	1060 (88.3)	134 (11.4)	6 (0.5)
Deep orange tubers	1134 (94.5)	57 (4.7)	9 (0.7)
Other vegetables	61 (5.08)	143 (11.9)	996 (83.0)
Citrus fruits	952 (79.3)	223 (18.5)	25 (2.1)
Other fruits	910 (75.8)	271 (22.5)	19 (1.5)
Legumes	181 (15.1)	425 (35.4)	594 (49.5)
Nuts and seeds	1051 (87.6)	121 (10.1)	28 (2.3)
Poultry	1179 (98.2)	21 (1.7)	0 (0)
Fish	1194 (99.5)	6 (0.5)	0 (0)
Whole grains	13 (1.1)	44 (3.6)	1143 (95.2)
Liquid oils	130 (10.8)	31 (2.6)	1039 (86.5)
Low fat dairy	939 (78.2)	210 (17.5)	51 (4.2)
Eggs	951 (79.2)	236 (19.6)	13 (1.1)
**Unhealthy Food groups**	**Never or 1/wk**	**2–4/wk**	**5–7/wk or**≥ **1/day**
White roots and tubers	35 (2.92)	461 (38.4)	704 (58.6)
Red meat	1111 (92.6)	89 (7.4)	0 (0)
Processed meat	9 (0.7)	219 (18.2)	972 (81)
Refined grains and baked goods	727 (60.6)	361 (30.1)	112 (9.3)
Sugar-sweetened beverages	24 (2)	191 (15.9)	985 (82.1)
Sweets and ice cream	81 (6.7)	348 (29)	771 (64.2)
High fat dairy	927 (77.2)	230 (19.1)	43 (3.6)
Juice	5 (0.4)	65 (5.4)	1130 (94.1)
Fried foods eaten away from home	42 (3.5)	301 (25.1)	857 (71.4)

The overall stunting prevalence was 8.17% with 95% CI: 6.74–9.85%. From the total stunted cases 61(62.24%) are males and 37(37.76%) female school adolescents.

### Factors associated with stunting among school adolescents

Sex of school-attending adolescents were significantly associated with stunting status of school adolescents. Males were 1.95 times more likely to be stunted compared to female adolescents with 95% CI: 1.11,3.39 in the final model controlling other factors (Table 3).

**Table 3:** Factors to stunting, Urban school-attending adolescents in Addis Ababa, 2019 Ethiopia

Characteristic	Stunting Status		Crude OR (95% CI)	Adjusted OR (95% CI)
	Not stunted	Stunted		
**Gender**				
Female	620	37	1	1
Male	482	61	1.99 (1.437,2.768) ***	1.95 (1.11,3.39) **
**Grade**				
Garde 5	280	20	1	1
Grade 6	273	27	1.38 (0.817,2.231)	0.72 (0.30,1.71)
Garde 7	276	24	1.2 (0.70,2.057)	0.49 (0.18,1.32)
Grade 8	273	27	1.38 (0.814,2.239)	0.33 (0.11,0.98) **
**Age of Adolescents**				
10 years	50	1	1	1
11 Years	150	10	3.187 (0.507,20.039)	2.76 (0.32,23.82)
12 Years	223	17	3.612 (0.516,25.287)	4.74 (0.56,40.22)
13 Years	282	13	2.247 (0.283,17.879)	3.58 (0.39,33.03)
14 Years	234	36	6.799 (1.094,42.259)	16.19 (1.74,150.82)
**Mother/female guardian occupation**				
Private	373	34	1	1
Government worker	208	30	1.509 (0.824,2.762)	1.45 (0.73,2.87)
Unemployment	45	1	0.260 (0.038,1.764)	0.39 (0.05,3.18)
Homemaker	368	23	0.704 (0.448,1.106)	0.64 (0.33,1.25)
**Fathers’/male guardian educational status**				
No schooling	203	22	1	1
Primary	395	35	0.832 (0.571,1.212)	1.32 (0.63,2.77)
Secondary	234	17	0.693 (0.368,1.304)	0.93 (0.40,2.15)
Technical and University	63	4	0.611 (0.267,1.394)	1.16 (0.32,4.17)
**Cruciferous vegetables**				
Never or 1/wk	923	88	1	1
2–4/wk	175	9	0.562 (0.304,1.037)	0.62 (0.27,1.44)
5–7/wk or > 1/day	4	1	2.297 (0.0558,9.459)	2.09 (0.34,13.07)
**Other fruits**				
Never or 1/wk	828	82	1	1
2–4/wk	257	14	0.573 (0.331,0.991)	0.58 (0.27,1.24)
5–7/wk or > 1/day	17	2	1.168 (0.288,4.738)	1.19 (0.26,5.51)
**Legumes**				
Never or 1/wk	171	10	1	1
2–4/wk	392	33	1.405 (0.777,2.543)	1.34 (0.56,3.23)
5–7/wk or > 1/day	539	55	1.676 (0.880,3.191)	1.72 (0.74,3.99)
**Liquid oils**				
Never or 1/wk	125	5	1	1
2–4/wk	30	1	0.839 (0.122,5.748)	0.98 (0.09,9.82)
5–7/wk or > 1/day	947	92	2.302 (0.9147,5.795)	2.21 (0.74,6.59)
**Sweets and ice cream**				
Never or 1/wk	79	2	1	1
2–4/wk	321	27	3.142 (0.700,14.096)	1.19 (0.25,5.64)
5–7/wk or > 1/day	702	69	3.624 (0.898,14.615)	1.83 (0.41,8.11)

NB: ***= p < .001, **= p < .01, *= p < .05

### Factors associated to thinness among school adolescents

Multivariable multinomial logistic regression indicates that gender and grade were statistically significant with thinness status of school adolescents. The associations between consumption of citrus fruits, low fat dairy and white roots and tubers school adolescents’ thinness status were statistically significant. Except white roots and tubers all food groups (Citrus fruits, Low fat dairy) were under healthy diet food group category. Thinness compared with consumption of Low-fat dairy from 2 to 4 times per week compared to non-consumption, the relative risk for thinness to normal would be expected to increase by a factor of 1.66. While consumption of citrus fruits and white roots and tubers 2–4 times per week compared to non-consumption or once per week the risk for thinness decreased by a factor of 0.52 and 0.28 respectively compared to normal school adolescents’ nutritional status (Table 4).

**Table 4:** Specific food item factors to thinness among Urban school-attending adolescents in Addis Ababa ,2019 Ethiopia, (*n* = 1200)

Specific food item frequency of consumption by adolescents	Thinness		Crude IRR (95% CI)	Adjusted IRR (95% CI)
	Normal	Thin		
**Gender**				
Female	501	68	1	1
Male	446	84	1.387 (0.98, 1.957)	1.45 (1.06,1.99) **
**Grade**				
Garde 5	250	32	1	1
Grade 6	228	47	1.61 (0.993,2.612)	1.80 (1.03, 3.15) **
Garde 7	243	30	0.965 (0.569,1.636)	1.09 (0.70,1.69)
Grade 8	226	43	1.486 (0.909,2.43)	1.56 (0.94,2.61)
**Fathers’/male guardian educational status**				
No schooling	178	27	1	1
Primary	336	63	1.236 (0.76,2.01)	1.19 (0.73,1.97)
Secondary	206	26	0.832 (0.468,1.478)	0.76 (0.42,1.38)
Technical and University	48	11	1.511 (0.6993.263)	1.49 (0.738,3.03)
**Deep orange vegetables**				
Never or 1/wk	675	102	1	1
2–4/wk	261	46	1.16 (0.91,1.49)	1.05 (0.72,1.52)
5–7/wk or > 1/day	11	4	2.41 (0.79,7.36)	1.81 (0.63,5.18)
**Deep orange tubers**				
Never or 1/wk	899	139	1	1
2–4/wk	44	9	1.32 (0.53,3.31)	1.14 (0.38,3.43)
5–7/wk or > 1/day	4	4	6.46 (1.57,26.55)	6.05 (0.52,70.78)
**Citrus fruits**				
Never or 1/wk	740	131	1	1
2–4/wk	189	17	0.51 (0.29,0.87)	0.52 (0.26,0.99)**
5–7/wk or > 1/day	18	4	1.25 (0.60,2.61)	1.63 (0.64,4.16)
**Low fat dairy**				
Never or 1/wk	752	107	1	1
2–4/wk	158	35	1.56 (1.08,2.24)	1.66 (1.07,2.58)**
5–7/wk or > 1/day	37	10	1.89 (0.91,3.97)	1.85 (0.76,4.49)
**Egg**				
Never or 1/wk	757	120	1	1
2–4/wk	181	28	0.97 (0.67,1.42)	0.84 (0.48,1.48)
5–7/wk or > 1/day	9	4	2.80 (1.14,6.91)	2.25 (0.65,7.71)
**White roots and tubers**				
Never or 1/wk	22	10	1	1
2–4/wk	347	68	0.43 (0.20,0.91)	0.44 (0.15,1.30)
5–7/wk or > 1/day	578	74	0.28 (0.13,0.59)	0.28 (0.09,0.83)**
**Refined grains and baked goods**				
Never or 1/wk	563	103	1	1
2–4/wk	294	34	0.63 (0.41,0.97)	0.69 (0.45,1.05)
5–7/wk or > 1/day	90	15	0.91 (0.44,1.86)	0.98 (0.44,2.18)
**High fat dairy**				
Never or 1/wk	743	107	1	1
2–4/wk	172	37	1.49 (1.03,2.16)	0.99 (0.61,1.63)
5–7/wk or > 1/day	32	8	1.74 (0.65,4.62)	1.09 (0.34,3.55)
**Fried foods eaten away from home**				
Never or 1/wk	35	3	1	1
2–4/wk	242	29	1.39 (0.41,4.73)	0.95 (0.27,3.33)
5–7/wk or > 1/day	670	120	2.09 (0.68,6.43)	1.79 (0.56,5.72)

NB: ***= p < .001, **= p < .01, * =p < .05

## Discussion

The prevalence of stunting and thinness were 8.17% and 12.67% respectively among school-attending adolescents in Addis Ababa. In this finding there was no association between adolescents stunting and thinness with the overall GDQS. Males and older school-attending adolescents were significantly associated with stunting status. Consumption of citrus fruits and white roots tubers were positively associated to thinness. While consumption of low-fat dairy for 2–4 times per week was positively associated with thinness status of school adolescents.

The prevalence of stunting in this study was low compared to previous studies among in school adolescent [6–8]. This low stunting prevalence in our study may be due to this study conducted in urban settings [8] .In urban setting families have an opportunity to get more media exposure compared to rural setting [37]. In addition, reginal variation due to sociodemographic and climatic variation between regions in Ethiopia may be the cause of the difference compared to previous studies among school adolescents [11]. This implies the need of different setting contextual action for the implementation of nutritional intervention program for adolescent population. Boys are more stunted than girls in our finding. Our finding is supported by similar study conducted in Ethiopia [38]. In Ethiopia culturally boys had more free time to participate in outdoor activities compared to adolescent girls; adolescent boys physical activity may influence their body weight [39].

The prevalence of thinness in Our finding is lower than studies conducted in the northern 29% [8] and 14.9% [14] and 15% [7] southern part of Ethiopia. This may be due to different sociodemographic characteristics and adolescents from rural settings which are more likely to be involved in activities which need more energy expenditure [40] and different cultural characteristics in the rural setting [41]. While our finding was higher than compared to previous studies conducted in Ethiopia 8.8% [6], 4.9% [13], and 5.2% [4]. The high prevalence in the study setting needs further investigation.

The male school-attending adolescents were more likely to be stunted than female adolescents; which is consistent to previous studies [13, 42]. In Ethiopia, boys have more free time to participate in outdoor physical activities compared to adolescent girls; which influence their body weight [12]. School adolescents age increases the probability of stunted increased [11], while in this study there is no statistical significance between adolescents age to stunting. This may be due to available difference between boys and girls towards their growth spurt age [43].

The overall GDQS was not statically significant with school adolescents stunting and thinness. In this study the mean overall GDQS food group score was 19.99 while in non-lactating and non-pregnant women in India overall GDQS food group score was 24 [25]. The overall mean difference GDQS food group score value between in this school adolescent group and non-lactating and non-pregnant women may be due to age of the two populations. This indicate that the need for further food quality measurement for adolescents.

Healthy food groups like citrus fruits, deep orange tubers, low fat dairy are an important diet components for humans [44]. In this study indicated that school adolescents’ thinness has association with consumption of citrus fruits, Low fat dairy and white roots and tubers. Almost 80% adolescents did not eat for the past seven days either citrus fruits or low-fat dairy products. In the other way 58% and 94% of adolescents take unhealth food groups like white root tubers and Juice respectively. This is an indication of malpractice for the frequency of consumption of health food group. Low-fat dairy products are reduced or naturally low-fat dairy products (≤ 2% milk fat) [45]. The frequency and consumption of healthy diets like citrus fruits and low-fat dairy products increased the overall GDQS score. While Consumption of unhealth food group like White roots and tubers has decreased the overall GDQS score. This inverse finding may be due to adolescent population difference compared to adult population. In addition, it indicated that the need for well-designed strong design to implement for adolescent population.

Some of the limitations of this study include social desirability bias leads to positively distorting dietary habit of adolescents. The study focused only for government schools and not including private schools in which stunting and thinness are not considered issue [32]. Consumption of certain food groups were considered as a wealthier household status in the community. So, this misconception and attitude of the respondent may increase the Global dietary quality score for school adolescents. Secondly, recall bias may underestimate the type of food consumed leading to lower dietary scores.

## Conclusion

This study revealed that the prevalence of thinness and stunting was high among urban school attending adolescents. The overall GDQS food score has no association with stunting and thinness status of school adolescents’ Nutritional interventions need to consider health food consumption. Consider the need for dietary quality, gender focused promotion interventions through effective behaviour change communications.

## Data Availability

The data that support the findings of this study are available from Addis Continental Institute of Public Health, but restrictions apply to the availability of these data, which were used under license for the current study, and so are not publicly available. Data are however available from the authors upon reasonable request and with permission of Addis Continental Institute of Public Health.

## References

[1] Das JK, Salam RA, Thornburg KL, Prentice AM, Campisi S, Lassi ZS (2017). Nutrition in adolescents: physiology, metabolism, and nutritional needs. Ann N Y Acad Sci.

[2] Ivers LC, Cullen KA (2011). Food insecurity: special considerations for women1234. Am J Clin Nutr.

[3] Manyanga T, El-Sayed H, Doku DT, Randall JR. The prevalence of underweight, overweight, obesity and associated risk factors among school-going adolescents in seven african countries. BMC Public Health. 2014;14:887. 10.1186/1471-2458-14-887 PMC4158085 25168589

[4] Teferi DY, Atomssa GE, Mekonnen TC. Overweight and Undernutrition in the Cases of School-Going Adolescents in Wolaita Sodo Town, Southern Ethiopia: Cross-Sectional Study. J Nutr Metab. 2018;2018:e8678561. 10.1155/2018/8678561 PMC5896243 29785306

[5] Degarege D, Degarege A, Animut A (2015). Undernutrition and associated risk factors among school age children in Addis Ababa, Ethiopia. BMC Public Health.

[6] Kahssay M, Mohamed L, Gebre A. Nutritional Status of School Going Adolescent Girls in Awash Town, Afar Region, Ethiopia. J Environ Public Health. 2020;2020:e7367139. 10.1155/2020/7367139 PMC7054789 32148529

[7] Gagebo DD, Kerbo AA, Thangavel T. Undernutrition and Associated Factors among Adolescent Girls in Damot Sore District, Southern Ethiopia. J Nutr Metab. 2020;2020:5083140. 10.1155/2020/5083140 PMC7350169 32685206

[8] Arage G, Assefa M, Worku T. Socio-demographic and economic factors are associated with nutritional status of adolescent school girls in Lay Guyint Woreda, Northwest Ethiopia. SAGE Open Med. 2019;7:2050312119844679. 10.1177/2050312119844679 PMC6469276 31019699

[9] Tamrat A, Yeshaw Y, Dadi AF. Stunting and Its Associated Factors among Early Adolescent School Girls of Gondar Town, Northwest Ethiopia: A School-Based Cross-Sectional Study. BioMed Res Int. 2020;8850074. 10.1155/2020/8850074 PMC7604580 33163537

[10] Gurzkowska B, Kułaga Z, Litwin M, Grajda A, Świąder A, Kułaga K (2014). The relationship between selected socioeconomic factors and basic anthropometric parameters of school-aged children and adolescents in Poland. Eur J Pediatr.

[11] Hailegebriel T (2020). Prevalence and determinants of stunting and Thinness/Wasting among Schoolchildren of Ethiopia: a systematic review and Meta-analysis. Food Nutr Bull.

[12] Abera M, Hardy-Johnson P, Abdissa A, Workicho A, Ali R, Weller S, et al. Social, economic and cultural influences on adolescent nutrition and physical activity in Jimma, Ethiopia: perspectives from adolescents and their caregivers. Public Health Nutrition. 2021;24(16):5218–26. 10.1017/S1368980020001664. 10.1017/S1368980020001664 PMC10195353 32727633

[13] Zemene MA, Engidaw MT, Gebremariam AD, Asnakew DT, Tiruneh SA. Nutritional status and associated factors among high school adolescents in Debre Tabor Town, South Gondar Zone, northcentral Ethiopia. BMC Nutr. 2019;5:43. 10.1186/s40795-019-0306-7 PMC7050895 32153956

[14] Mengesha DK, Prasad RPCJ, Asres DT. Prevalence and Associated Factors of Thinness among Adolescent Students in Finote Selam Town, Northwest Ethiopia. Sci World J. 2020;e9170301. 10.1155/2020/9170301 PMC7288196 32565753

[15] Yazdi Feyzabadi V, Keshavarz Mohammadi N, Omidvar N, Karimi-Shahanjarini A, Nedjat S, Rashidian A (2017). Factors Associated with unhealthy snacks Consumption among Adolescents in Iran’s schools. Int J Health Policy Manag.

[16] Salvy S-J, Elmo A, Nitecki LA, Kluczynski MA, Roemmich JN (2011). Influence of parents and friends on children’s and adolescents’ food intake and food selection123. Am J Clin Nutr.

[17] Neumark-Sztainer D, Story M, Perry C, Casey MA. Factors influencing food choices of adolescents: findings from focus-group discussions with adolescents. J Am Diet Assoc. 1999;99(8):929-37. 10.1016/S0002-8223(99)00222-9 10450307

[18] Eddy KT, Hennessey M, Thompson-Brenner H (2007). Eating Pathology in East African Women: the role of media exposure and globalization. J Nerv Ment Dis.

[19] Keats EC, Rappaport AI, Shah S, Oh C, Jain R, Bhutta ZA (2018). The Dietary Intake and Practices of adolescent girls in low- and Middle-Income Countries: a systematic review. Nutrients.

[20] Dalwood P, Marshall S, Burrows TL, McIntosh A, Collins CE (2020). Diet quality indices and their associations with health-related outcomes in children and adolescents: an updated systematic review. Nutr J.

[21] Mirmiran P, Azadbakht L, Esmaillzadeh A, Azizi F (2004). Dietary diversity score in adolescents - a good indicator of the nutritional adequacy of diets: Tehran lipid and glucose study. Asia Pac J Clin Nutr.

[22] Bromage S, Batis C, Bhupathiraju SN, Fawzi WW, Fung TT, Li Y (2021). Development and validation of a novel food-based global Diet Quality score (GDQS). J Nutr.

[23] Fung T, Bromage S, Li Y, Bhupathiraju S, Batis C, Fawzi W, et al. A Global Diet Quality Index and Risk of type 2 diabetes in U.S. women. Curr Dev Nutr. 2020;4 Suppl 2:1401.

[24] Bromage S, Zhang Y, Holmes M, Fawzi W, Sachs S, Fanzo J, et al. A novel food-based Diet Quality score is Associated with nutrient adequacy and reduced Anemia among rural adults in ten african countries. Curr Dev Nutr. 2020;4 Suppl 2:1381.

[25] Matsuzaki M, Bromage S, Batis C, Fung T, Li Y, Deitchler M (2020). Validation of a New Instrument for assessing Diet Quality and its Association with Undernutrition and Non-Communicable Diseases for Women in Reproductive Age in India. Curr Dev Nutr.

[26] He Y, Fang Y, Bromage S, Fung TT, Bhupathiraju SN, Batis C (2021). Application of the global Diet Quality score in chinese adults to evaluate the double burden of nutrient inadequacy and metabolic syndrome. J Nutr.

[27] Bromage S, Andersen CT, Tadesse AW, Passarelli S, Hemler EC, Fekadu H (2021). The global Diet Quality score is Associated with higher nutrient adequacy, Midupper Arm circumference, venous hemoglobin, and serum Folate among Urban and rural ethiopian adults. J Nutr.

[28] Gonete KA, Tariku A, Wami SD, Akalu TY (2020). Dietary diversity practice and associated factors among adolescent girls in Dembia district, northwest Ethiopia, 2017. Public Health Rev.

[29] Meng L, Wang Y, Li T, van Loo-Bouwman CA, Zhang Y, Man-Yau Szeto I (2018). Dietary diversity and Food Variety in Chinese Children aged 3–17 years: are they negatively Associated with Dietary Micronutrient Inadequacy?. Nutrients.

[30] Ethiopia ICF, Central Statistical Agency - CSA/ (2017). Ethiopia Demographic and Health Survey 2016.

[31] Amaha ND (2020). Ethiopian progress towards achieving the global nutrition targets of 2025: analysis of sub-national trends and progress inequalities. BMC Res Notes.

[32] Gebreyohannes Y, Shiferaw S, Demtsu B, Bugssa G (2014). Nutritional status of adolescents in selected government and private secondary schools of Addis Ababa, Ethiopia. Int J Nutr Food Sci.

[33] Blössner M, Siyam A, Borghi E, Onyango A, De Onis M. WHO AnthroPlus for personal computers manual: software for assessing growth of the world’s children and adolescents. World Health Organ Geneva Switz; 2009.

[34] de Onis M, Onyango AW, Borghi E, Siyam A, Nishida C, Siekmann J (2007). Development of a WHO growth reference for school-aged children and adolescents. Bull World Health Organ.

[35] FAO., FHI 360. Minimum Dietary Diversity for Women: A Guide for Measurement. 2016.

[36] WHO. WHO AnthroPlus for personal computers Manual: Software for assessing growth of the world’s children and adolescents. 2009.

[37] Alemu TG, Muhye AB, Ayele AD (2021). Under nutrition and associated factors among adolescent girls attending school in the rural and urban districts of Debark, Northwest Ethiopia: a community-based comparative cross-sectional study. PLoS ONE.

[38] Melaku YA, Zello GA, Gill TK, Adams RJ, Shi Z (2015). Prevalence and factors associated with stunting and thinness among adolescent students in Northern Ethiopia: a comparison to World Health Organization standards. Arch Public Health Arch Belg Sante Publique.

[39] Abera M, Hardy-Johnson P, Abdissa A, Workicho A, Ali R, Weller S (2021). Social, economic and cultural influences on adolescent nutrition and physical activity in Jimma, Ethiopia: perspectives from adolescents and their caregivers. Public Health Nutr.

[40] Gebregyorgis T, Tadesse T, Atenafu A (2016). Prevalence of thinness and stunting and Associated factors among adolescent School Girls in Adwa Town, North Ethiopia. Int J Food Sci.

[41] Arage G, Assefa M, Worku T (2019). Socio-demographic and economic factors are associated with nutritional status of adolescent school girls in Lay Guyint Woreda, Northwest Ethiopia. SAGE Open Med.

[42] Melaku YA, Zello GA, Gill TK, Adams RJ, Shi Z (2015). Prevalence and factors associated with stunting and thinness among adolescent students in Northern Ethiopia: a comparison to World Health Organization standards. Arch Public Health.

[43] Tanner JM (1981). Growth and maturation during adolescence. Nutr Rev.

[44] Chandrasekara A, Josheph Kumar T (2016). Roots and tuber crops as Functional Foods: a review on Phytochemical Constituents and their potential health benefits. Int J Food Sci.

[45] Intake, –, Center for Dietary Assessment (2021). The global Diet Quality score: data Collection Options and Tabulation Guidelines.

