# Correction: Stunting and thinness in school-attending adolescents in Addis Ababa

**DOI:** 10.1186/s40795-023-00673-5

**Published:** 2023-01-17

**Authors:** Walelegn Worku Yallew, Amare Worku Tadesse, Ramadhani Abdallah Noor, Wafaie Fawzi, Yemane Berhane

**Affiliations:** 1 Addis Continental Institute of Public Health, Po. Box 196, Addis Ababa, Ethiopia; 2 Department of Infectious Diseases Epidemiology, London School of Hygiene and Tropical Medicine, London, UK; 3 United Nations Children’s Fund, Addis Ababa, Ethiopia; 4 Department of Nutrition, Harvard TH Chan School of Public Health, Boston, MA USA

**Correction: BMC Nutr 8, 159 (2022)**

**https://doi.org/10.1186/s40795-022-00653-1**

Following publication of the original article [1], the authors identified an error in the author name Ramadhani Abdallah Noor.

The incorrect author name is: Abdallah Noor

The correct author name is: Ramadhani Abdallah Noor

Further to this, affiliation 3 has been corrected to United Nations Children’s Fund, Addis Ababa, Ethiopia. The author group and affiliations have been updated above and the original article [1] has been corrected.
